# Using Dynamic Laser Speckle Imaging for Plant Breeding: A Case Study of Water Stress in Sunflowers

**DOI:** 10.3390/s24165260

**Published:** 2024-08-14

**Authors:** Sherif Bouzaouia, Maxime Ryckewaert, Daphné Héran, Arnaud Ducanchez, Ryad Bendoula

**Affiliations:** 1 ITAP, INRAE, Institut Agro, University of Montpellier, 34060 Montpellier, France; daphne.heran@inrae.fr (D.H.); arnaud.ducanchez@supagro.fr (A.D.); ryad.bendoula@inrae.fr (R.B.); 2 (LIRMM) Laboratory of Computer Science, Robotics and Microelectronics of Montpellier, Inria, (CNRS) National Center for Scientific Research, University of Montpellier, 34392 Montpellier, France; maxime.ryckewaert@inria.fr

**Keywords:** biospeckle, water stress, digital agriculture, phenotyping

## Abstract

This study focuses on the promising use of biospeckle technology to detect water stress in plants, a complex physiological mechanism. This involves monitoring the temporal activity of biospeckle pattern to study the occurrence of stress within the leaf. The effects of water stress in plants can involve physical and biochemical changes. Some of these changes may alter the optical scattering properties of leaves. The present study therefore proposes to test the potential of a biospeckle measurement to observe the temporal evolution in different varieties of sunflower plants under water stress. An experiment applying controlled water stress with osmotic shock using polyethylene glycol 6000 (PEG) was conducted on two sunflower varieties: one sensitive, and the other more tolerant to water stress. Temporal monitoring of biospeckle activity in these plants was performed using the average value of difference (AVD) indicator. Results indicate that AVD highlights the difference in biospeckle activity between day and night, with lower activity at night for both varieties. The addition of PEG entailed a gradual decrease in values throughout the experiment, particularly for the sensitive variety. The results obtained are consistent with the behaviour of the varieties submitted to water stress. Indeed, a few days after the introduction of PEG, a stronger decrease in AVD indicator values was observed for the sensitive variety than for the resistant variety. This study highlights the dynamics of biospeckle activity for different sunflower varieties undergoing water stress and can be considered as a promising phenotyping tool.

## 1. Introduction

In recent years, the development of new sensors has expanded the possibilities for crop management within the framework of digital agriculture or precision agriculture [1,2,3]. Sensors are developed to produce information for the monitoring of vegetation and some of them can be used for detecting biotic or abiotic stress events. This information helps us to act accordingly, similarly to the approach towards irrigation, nitrogen fertilization, or the onset of harvesting. In this field, optical sensors offer the benefit of a non-invasive and non-destructive approach for extracting information from vegetation [4,5].

More specifically, optical sensors can be used for studying the emergence of a disease [6,7], or a hydric stress [8,9]. Improving observational capabilities through the development of new sensors could offer a better understanding of the physiological phenomena involved.

Vegetation leaves are biological media with complex structures, and are considered to scatter light across a wide spectral range [10]. Moreover, when a heterogeneous medium is illuminated by a coherent light, an interference phenomenon called speckle occurs resulting from the light–matter interaction and from various optical paths taken by the light rays [11]. In the case of a living medium, the optical ray path evolves with time, due to particle mobility within the sample. In this case, the interference pattern is dynamic, and is designated as biospeckle [12].

Recently, biospeckle applications have emerged in the field of food [13] and agriculture [14]. These applications include the detection of diseased foliage [15], the monitoring of ageing and shelf life of fruits [14], fruit infestation [16], the monitoring of root growth [17], the detection of contaminated bean seeds [18], or access to the quality attributes of apples [19].

The use of biospeckle imaging, in particular the temporal monitoring of targeted parts of the plant, has proven to be appropriate for monitoring events such as the occurrence of abiotic [14,20,21] or biotic stress [22,23,24] and, more specifically, to detect water stress [25]. Indeed, the establishment of water stress within a plant is a complex process in which physiological mechanisms evolve gradually over time [26,27]. These evolutions, among other internal leaf changes, can be studied through the analysis of the leaf biospeckle activity in order to identify the onset of water stress.

To analyse biospeckle images, different approaches can be employed. Amongst these, graphical and numerical methods are often used [28]. Graphical methods allow for the identification of homogeneous areas inside the biospeckle images. The Fujii method represents a reference graphical method. It has proven its ability to produce high quality activity maps, thus highlighting the distribution of biospeckle activity throughout an analysed sample [29].

Another manner to analyse biospeckle data is to employ numerical methods [12,30]. Here, the AVD index is calculated for each measurement, thus enabling the temporal evolution of AVD values to be monitored. This method provides a detailed quantitative analysis of the dynamic changes in biospeckle patterns over time, offering insights into the biological activities of the studied samples.

The numerical method represents a means to quantify the level of change in the speckle patterns within a homogeneously identified area of interest, which can be initially identified using the graphical method. This activity level, measured over time, provides a quantitative measure of the internal dynamics of the sample area studied, enabling detailed analyses and comparisons between different samples and experimental conditions. The present work thus proposes to test the potential of a biospeckle measurement for observing the temporal evolution of a sunflower plant under water stress and for different varieties.

An experiment was conducted to simulate and control the water stress due to an osmotic shock on sunflowers, with polyethylene glycol 6000 (PEG) was introduced into the nutrient medium during the biospeckle measurements. The biospeckle activity of two varieties of sunflower, one sensitive and the other tolerant to water stress, was monitored and compared throughout the whole duration of the experiment, using both graphical and numerical methods. Additionally, control measurements were also conducted on the sensitive variety, without stress, to better understand the effects of the osmotic shock on the varieties.

## 2. Materials and Methods

### 2.1. Experimental Design

An experimental design was developed in order to compare two sunflower genotypes under osmotic stress. The sunflower seeds was provided by the company Innolea, Mondonville, France. These genotypes were chosen for their resistance to drought, with one being sensitive (variety S) and the other more tolerant (variety T). The sunflower plants were grown under hydroponic conditions in a greenhouse at INRAE, France. The hydroponic solution was composed of distilled water and Murashige and Skoog medium (MS), with a 2.5 g/L dilution. Conditions of illumination were similar for each pot, consisting in a day–night cycle of 16 h/8 h. The greenhouse was equipped with multispectral lighting (450 nm, 560 nm, 660 nm, 730 nm and 6000 °K) controlled by Herbro automaton (GreenHouseKeeper), while the PAR on canopy was equal to 210 μmol/m^2^/s.

Seeds from the two genotypes were grown simultaneously. It took four to five weeks between seed germination and the first measurement. The plants were measured in a laboratory when they reached the stage between the fourth or fifth floral nodes on a sufficiently developed and healthy leaf, prior to the development of the floral bud.

**Figure 1:**
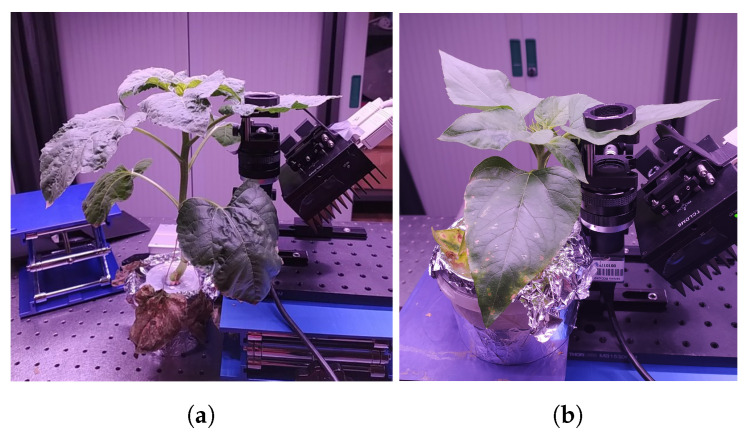
Pictures of plants on the measurement bench (**a**) variety T, and (**b**) variety S.

The plant was measured in a laboratory. The day/night cycle in the laboratory was 14 h/10 h, and lighting was provided by two LED strips resulting in PAR ranging from 90 to 120 μmol/m^2^/s. For each studied sunflower leaf, an area was delineated using a 25 mm-diameter magnetic holder around 3 cm from the base of the blade. This area continuously monitored during the various acquisitions (see Figure 1). Polyethylene glycol 6000 (PEG) was used to induce a water deficit [31], with a concentration of 200 g/L of MS solution. This solution was introduced as a nutrient medium for the plant, during the third day of measurements. In total, measurements were acquired for each leaf over eight days.

Two realisations were carried out for both variety S and variety T. In addition, two realisations were carried out for variety S under normal conditions as a control.

A reference measurement was made using a white paper card as an inert object. The measurement information is summarised in Table 1 below.

### 2.2. Biospeckle Acquisitions

**Figure 2:**
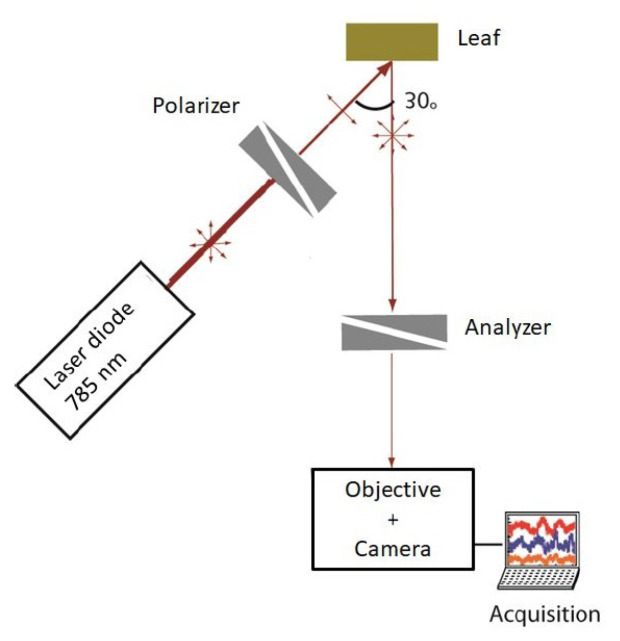
Diagram of the biospeckle acquisition system.

The optical setup used for biospeckle measurements is illustrated schematically in Figure 2. The setup was provided by Thorlabs, Bergkirchen, Germany. A 785 nm (L785P100) laser diode was used to excite the sample. At this wavelength, plants exhibited a reflectance plateau, partly attributed to the low absorption of light by plant pigments and also to the scattering effect of the leaf structure [32]. This characteristic promotes maximum light scattering. This is particularly suitable for the use of biospeckle, which relies on the analysis of scattered light. The laser diode was adjusted to set the laser spot around 25 mm-diameter at the sample surface.

A grid polarizer (WP12L-UB) was mounted between the laser diode and the sample to set a p-polarization. An analyser (WP25M-UB) was mounted in front of the camera (CMOS, DCC3240M) in order to measure only backscattered speckle in s- polarization, while eliminating specular light. An imaging objective f = 25 mm, open at F/16, was mounted in front of the camera with a 10 mm spacer, in order to image the leaf on the sensor.

The frame rate was set to 14.4 fps and the integration time of the camera was set to 12 ms in order to avoid blur on the image due to particle motion in the biological sample. Measurements were taken every twenty minutes. For each acquisition, a collection of forty frames was taken in a row. This sampling method, based on the acquisition of forty frames, was adopted to achieve an optimal balance between the image storage capacity and the effective capture of optical interference tracking over time.

### 2.3. Data Analysis

In order to monitor the temporal behaviour of the samples, biospeckle images were processed in two steps. The first step was to identify a region of interest (RoI) on the leaf, characterised by a high level of activity. This step was based on the Fujii method [33,34]. The second step was to calculate the average value of difference (AVD) indicator [12] on the identified RoI and to study its temporal evolution.

#### 2.3.1. Activity Maps to Identify a Region of Interest

The graphical method. referred to as the Fujii [33,34], was used to select a RoI. This method can be distinguished by ability to provide spatial resolution in speckle activity analysis. This graphical indicator was calculated for each biospeckle acquisition (comprising 40 frames) using the following equation:

(1) $$AD=\sum_{k=1}^{N}\frac{|I_{k}-I_{k-1}|}{I_{k}+I_{k-1}}$$

where $I_{k}$ corresponds to the intensity matrix of the image at frame *k*, and *N* is the number of frames per image.

The outcome of this graphical method can be seen as a map of activity of the sample. Then, masks were obtained by adjusting a threshold value on Fujii activity maps to select a RoI with a high level of activity. A mask was thus created for each.

#### 2.3.2. AVD Indicator Computation

The average value of difference (AVD) indicator was calculated on the selected RoI, for each biospeckle acquisition. To do so, pixels contained in the RoI were used for building the Time History Speckle Pattern (THSP) matrix [35,36]. The THSP method tracks the temporal evolution of speckle patterns. It is particularly effective to detect dynamic changes in samples, such as biological tissues. The THSP method is especially useful to observe processes that unfold over time, providing insights into dynamic biological or inert material. In this experiment, the THSP matrix was an M×N matrix, where *M* is the number of pixels in the RoI and *N* is the number of frames. It contains the successive intensity values of the 40 frames of a biospeckle acquisition, for each pixel of the RoI. For each THSP matrix, a co-occurrence matrix (COM) was calculated, representing the transition histogram of the intensity. Thus, for a transition of intensity from a level *i* to a level *j*, the corresponding value of matrix COM(i,j) was computed as follows:

(2) $$COM\left(i,j\right)=\sum_{m=1}^{M}\sum_{n=1}^{N-1}\left\{\begin{array}{cc} 1, & \mathrm{if}THSP(m,n)=i\mathrm{and}THSP(m,n+1)=j\\ 0, & \mathrm{otherwise} \end{array}\right.$$

Two approaches to the normalisation of the COM matrix were used. The first approach $M_{ij,1}$ applied the normalisation proposed by [37] (Equation (3)) and the second approach $M_{ij,2}$ applied the normalisation proposed by [12] (Equation (4)).

(3) $$M_{ij,1}=\frac{COM(i,j)}{\sum_{lm}COM\left(l,m\right)}$$

(4) $$M_{ij,2}=\frac{COM(i,j)}{\sum_{m}COM\left(i,m\right)}$$

As described in “A practical guide to biospeckle laser analysis” [38], AVD indicators can be computed using different normalisations. Here, AVD1 used $M_{ij,1}$ normalisation (3), while AVD4 uses $M_{ij,2}$ normalisation (4).

(5) $$AVD1=\sum_{ij}M_{ij,1}\left|i-j\right|$$

(6) $$AVD4=\sum_{ij}M_{ij,2}\left|i-j\right|$$

These different indices were calculated with Matlab R2020a thanks to the Bio-Speckle Laser Tool Library [39].

Finally, the curves representing the temporal evolution of the AVD were smoothed using the Savitzky–Golay algorithm to reduce the residual noise.

**Figure 3:**
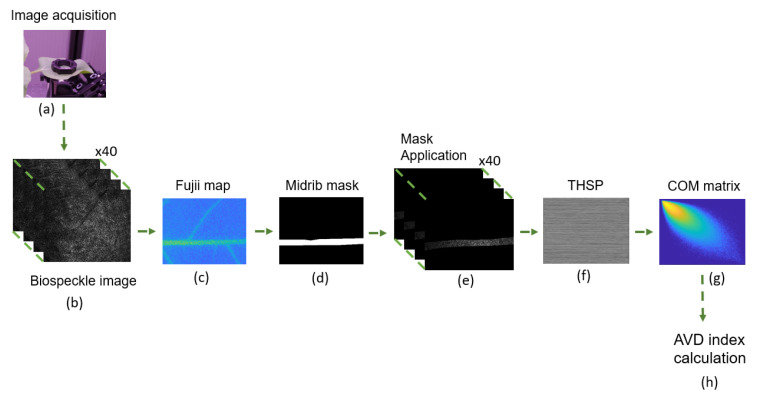
For each realisation and acquisition (**a**), 40 biospeckle images are captured (**b**). A Fujii activity map is generated from these images (**c**), and a mask is created (**d**) based on a threshold on this map. This mask is then applied to the 40 biospeckle images to obtain the midrib segmentation (**e**). A THSP matrix is computed (**f**) for each pixel of the segmented midrib, and a COM matrix is computed (**g**). Finally, AVD index is calculated from the COM matrix (**h**).

All of the analysis methods described above are combined into a processing chain. It starts from data acquisition and results in the AVD index. The complete processing chain is described by Figure 3.

#### 2.3.3. Signal Analysis

For the control plants and inert object, the averages of AVD1 values and AVD4 values were calculated for both night-time and day-time periods in order to calculate the signal-to-noise ratio (SNR). AVD1 and AVD4 values of control plants were considered to be signals while AVD1 and AVD4 values of inert object were assimilated to noise (7).

(7) $$SNR=\left(\frac{Signal}{Noise}\right)$$

## 3. Results

### 3.1. Identification of a Homogeneous Area of Interest

**Figure 4:**
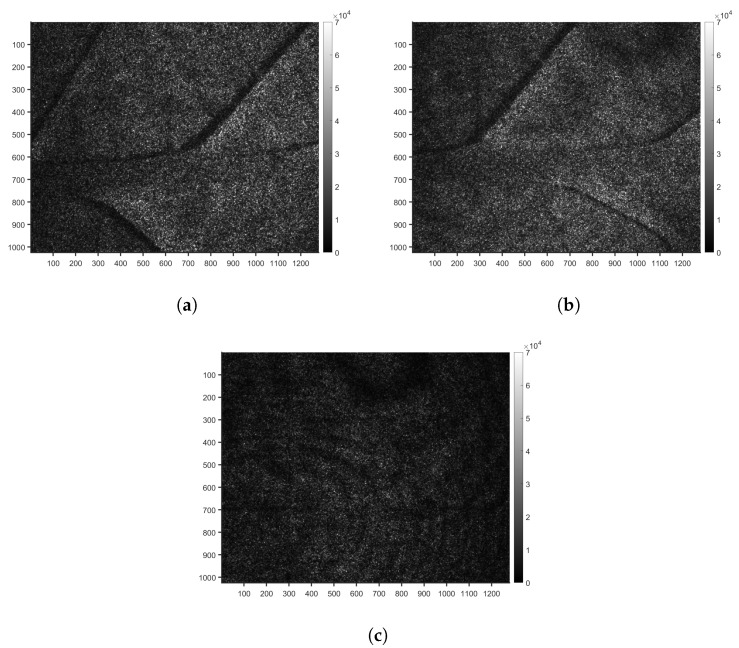
Raw biospeckle images at the beginning of the experiment for two control plants of the S variety, (**a**) realisation 5 and (**b**) realisation 6 and on an inert object (**c**) realisation 7. The colour bar represents greyscale intensity.

Figure 4a,b illustrates the speckle pattern observed at the beginning of the experiment for two control plants of the S variety for the realisations 5 and 6 (Table 1). These patterns are characterised by a random distribution of bright and dark spots, resulting from the random scattering of light waves in phase or out of phase, emitted by the leaf surface. For both realisations, the leaf veins could be distinguished in the images. The choice of the integration time was optimised in order to avoid pixel saturation and speckle pattern blur.

**Figure 5:**
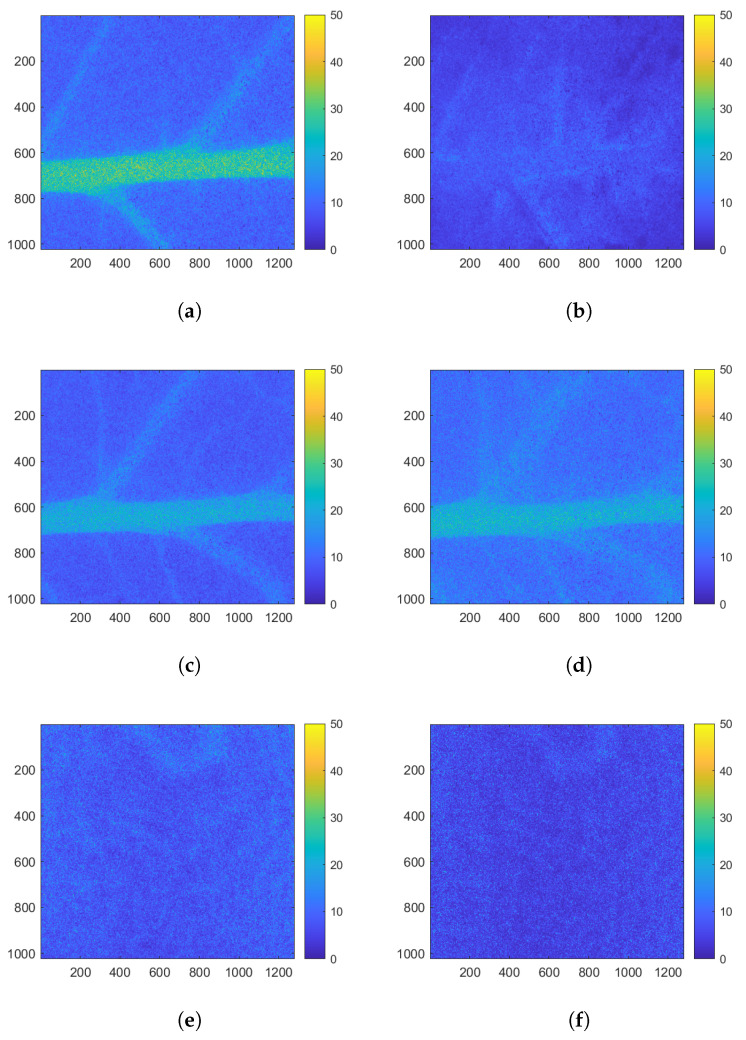
Biospeckle activity maps calculated by Fujii method at the beginning of experiment for realisations 4 (**a**), 5 (**c**), and 7 (**e**) (inert object). At the end of experiment, these are, respectively, (**b**,**d**,**f**). The colour bar represents the intensity of biospeckle activity across 40 biospeckle images.

From the raw biospeckle images of the two realisations of the S variety and the inert object, biospeckle activity maps were calculated using the Fujii method (see Equation (1)). Biospeckle activity maps for realisations 5 and 6 and the inert object at the beginning of the experiment are illustrated by Figure 5a,c,e, respectively, and at the figures at the end of the experiment in Figure 5b,d,e.

At the beginning of the experiment, the Fujii maps present values ranging from 0 to 50%. The highest values can be observed within the leaf veins, particularly in the midrib, for realisation 4 (Figure 5a) and 5 (Figure 5c). At the level of the limbus, values are much lower with values lower (less than 20%). For the inert object (Figure 5e), values are homogeneous across the entire image.

Transportation of water, mineral or glucose takes place in the midribs [40] photosynthetic processes and biogeochemical reactions occur in the limbus [41]. This high activity rate depicted by the Fujii maps corresponds to activity in the midrib, which is greater than that in the limb. These observations corroborate those previously described by [42,43].

At the end of the experiment, the values in the leaf veins for realisation 4 (Figure 5d) were no longer visible, whereas they remained clearly visible for realisation 5 (Figure 5b). For the inert object (Figure 5f), the levels on the Fujii map were constant. For realisation 4, PEG was introduced into the plant nutrient medium, causing stress. This stress was illustrated as by a reduction in the values on the Fujii map, particularly along the midrib of the leaf.

The Fujii method revealed that the veins of the leaves, particularly the midrib, had a high level of activity relative to the rest of the leaf. When PEG was applied, this activity was evenly disturbed evenly throughout this zone which, therefore, contains significant information on the evolution of the leaf status. Thanks to the homogeneity of this zone, temporal monitoring is ideal, thus enabling precise quantification of the activity of both types of studied leaves. For this reason, this specific area was chosen for defining masks, in order to segment a region of interest on which the rest of the study could be focused.

**Figure 6:**
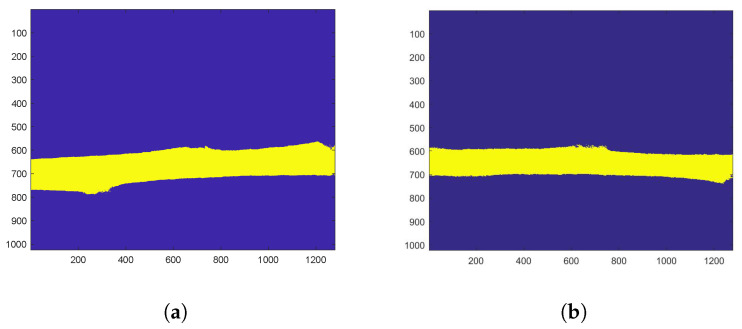
Masks obtained for isolating the midrib for (**a**) realisation 4 and (**b**) realisation 5 of the S variety. The masks are calculated using a threshold of the Fujii values.

During the rest of the study, THSP, COM and AVD values were calculated on the mask-corresponding pixels (Figure 6), which are the midrib pixels.

### 3.2. Midrib Biospeckle Activity

#### 3.2.1. Co-Occurrence Matrices

**Figure 7:**
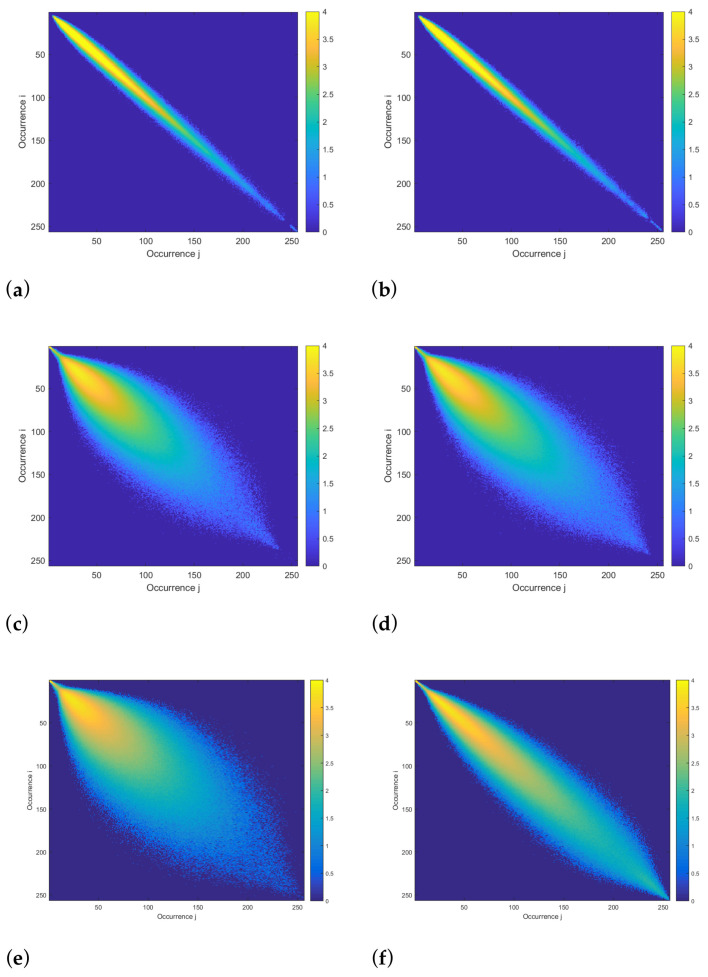
COM matrices of midrib pixels: (**a**,**b**) inert object at the beginning and end of the experiment. (**c**,**d**) realisation 5 at the beginning and at the end of the experiment, respectively, and (**e**,**f**) realisation 4 at the beginning and end of the experiment, respectively. The colour bar represents the identical occurrences of an intensity level “i” at level “j” in two consecutive intensity samples within a THSP matrix. Intensity values are displayed in log-scale.

Figure 7 represents different COM matrices derived from THSP matrices at the beginning of the experiment, for realisation 3 (Figure 7e) and 5 (Figure 7c) of the S variety, particularly focusing on their midribs and the inert object (Figure 7a).

Points located along the diagonal of the COM matrix correspond to pixels where the intensity value did not change from one frame to another. Conversely, points outside of the diagonal indicate intensity changes from one frame to another. The colour gradation indicates the co-occurrence frequency of these values.

Regarding realisation 5 (Figure 7c) and the inert object (Figure 7b), no changes were observed in the COM matrix between the beginning and end of the experiment, thus corroborating previous observations made on the Fujii maps (Figure 7d,f). These results could be explained by the fact that realisation 5 was used as a control experiment, without PEG addition. Under these conditions, the plant was not subjected to any stress, and therefore no evolution of speckle patterns in the midrib was observable. As for the inert object, only the noise associated with the measurement instrumentation was measured, namely source variations or ambient vibration. It is noteworthy that, when compared with realisations on plants, the COM matrix associated with the inert object has presented dispersion around the matrix diagonal. These difference is attributed to the internal activity of the midrib which induces a high level of biospeckle activity and, therefore, a greater dispersion around the matrix diagonal.

At the beginning of the experiment, for realisation 4 (Figure 7e), a strong dispersion of intensity values on either side of the COM matrix diagonal was observed. This significant dispersion appeared to be of the same order as observed for realisation 5. For these two realisations, the matrix dispersion suggested a healthy biological activity within the midrib. This is perfectly normal, since the plants were healthy at the beginning of the experiment.

At the end of the experiment, for realisation 4 (Figure 7f), a lesser dispersion of the COM matrix was observed. This decrease indicated fewer changes in pixel intensity from one biospeckle frame to another. This reduction occurred following the addition of PEG. The introduction of PEG induced stress in the plant, resulting in a reduction in biological activity. This reduced activity, and hence the stress on the plant, was clearly visible with the COM matrix.

It appeared, therefore, that PEG causes a reduction in the internal activity of the midrib, and this was observable throughout the COM matrices. However, since neither the co-occurrence matrices nor the Fujii maps provided precise quantitative information on the biological activity of the leaf, our understanding of the precise state and physiological characteristics of the studied area remain limited.

#### 3.2.2. AVD Temporal Evolution

**Figure 8:**
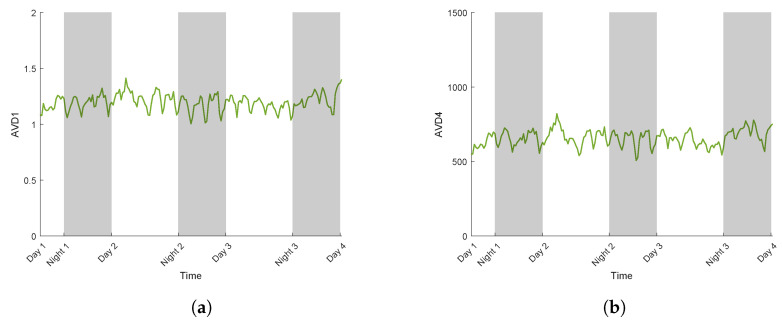
AVD index calculated on an inert object with the normalised COM matrix $M_{ij,1}$ (AVD1) (**a**) and $M_{ij,2}$ (AVD4) (**b**).

The AVD index is calculated for each biospeckle acquisition over time, providing a temporal evolution of biospeckle activity. The following figures illustrate the AVD index values with normalisation $M_{ij,1}$ (Figure 8a) and $M_{ij,2}$ (Figure 8b), measured on an inert object over four days, with the day/night cycle described in the Materials and Methods section. AVD with the $M_{ij,1}$ normalisation index values range between 1 and 1.5 throughout the measurement (Figure 8a) and between 500 and 1000 with $M_{ij,2}$ normalisation (Figure 8b).

For both normalisations, the AVD index values were centred on a constant value, thus indicating that the differences between day and night were not significant. This implies that the day–night cycle does not affect the AVD values for an inert object. The present variations in AVD values followed a similar shape between both normalisations. The fluctuations in the AVD index observed between each acquisition could therefore be attributable to the noise produced by the measuring instrument (diode laser temperature and intensity controller) and environmental conditions.

**Figure 9:**
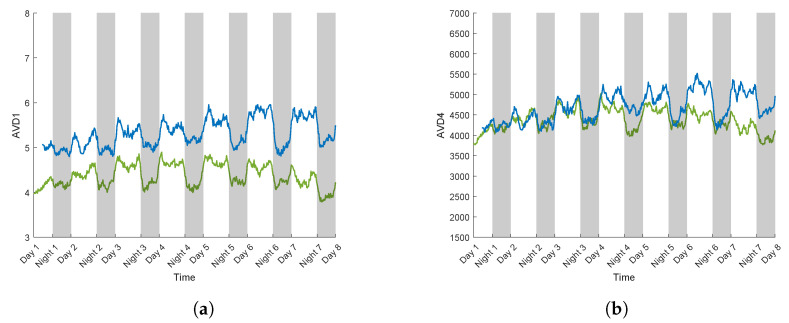
AVD index calculated on a control sunflower plant (realisation 5 in green and 6 in blue) of the S variety with the normalized COM matrix $M_{ij,1}$ (AVD1) (**a**), and $M_{ij,2}$ (AVD4) (**b**).

Figure 9a,b present the evolution of AVD index values on a sunflower of the S variety for realisation 5 and 6 (see Table 2), over eight days, without any PEG addition.

These measurements were used as control experiments. During measurements for realisation 5, AVD1 values oscillated within a range of 1.5 and AVD4 within 1300. For realisation 6, AVD1 values oscillated within a range of 1.5 and AVD4 within 1800. However, differences occurred in AVD values between day and night for both types of normalisation. AVD values were higher during the day than at night.

For realisation 5, daytime values could reach 5, while at night they were lower by 1 for AVD1. For AVD4, they reached 5000, while at night they decreased by 1000. For realisation 6, daytime values could reach 6, while at night they decreased by 1 for AVD1, and for AVD4, they reached 5500 while at night they decrease by 1500.

These results point to a significant influence of the diurnal and nocturnal phases on AVD values, thus potentially highlighting a physiological response of the sunflower during these phases [44]. Biospeckle activity was more intense during the day when it could be associated with more pronounced physiological phenomena. During the day, plants are known to undergo an increase in transpiration, photosynthesis [45], and water flow [46], especially sap flow [47]. On the contrary, at night, the activity slows down as plants enter a metabolic resting state in order to save their resources for the next diurnal cycle [48].

From the calculation of AVD1 and AVD4 during the day and night, signal-to-noise ratios were calculated between the control measurements and the inert object (see Equation (7)). The measurements on the inert object were considered as noise. Table 2 presents the values of these signal-to-noise ratios for realisations 5 and 6 during the day and night and for AVD1 and AVD4.

**Table 2:** SNR of the mean of AVD1 and AVD4 for day and night, with the control realisations 5 and 6 as signal and inert object (realisation 7) as noise.

Realisations	Days/Nights	SNR	
		AVD1	AVD4
5	Night	3.5	6.3
	Day	3.7	6.9
6	Night	4.3	6.7
	Day	4.6	7.5

For realisations 5 and 6, the SNR was higher when using AVD4. There was no variation in SNR from day to night. Higher SNR values could indicate improved measurement quality or enhanced an detectable signal of interest using the AVD4 indicator.

**Figure 10:**
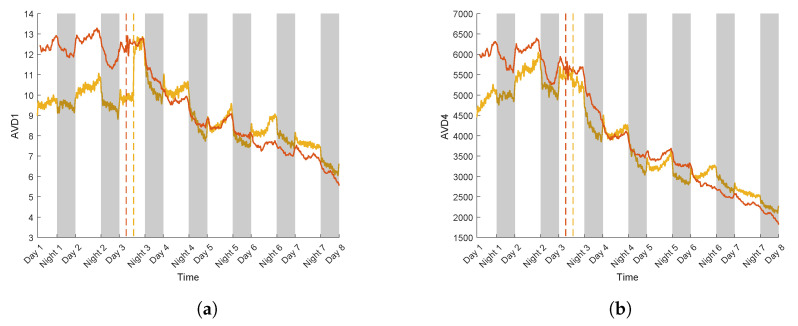
Biospeckle activity calculated by AVD. Colours yellow and orange refer, respectively, to realisations 3 and 4, while dotted lines correspond to PEG introduction with the normalized COM matrix $M_{ij,1}$ (**a**) and $M_{ij,2}$ (**b**).

Figure 10a,b illustrates the evolution of AVD1 and AVD4 for two realisations of the S variety where a water constraint protocol was applied (for realisation 3 and 4, see Table 1).

For both normalisations of AVD in the S variety under water stress (Figure 10a,b), differences in values between day and night were observed during the first two days of the experiment, with notably higher values of AVD during the day. This trend is consistent with that observed for the control realisations (see Figure 9a,b).

From day 3, a gradual decrease in AVD values was observed, continuing through day 8. For realisation 4, using AVD1, the values fell by 6, while with AVD4, there was a 3400 decrease. In the case of realisation 3, AVD values decreased by 3 with AVD1, while for AVD4 they fell by 3100.

There was an increase in AVD1 values on day 3 for realisation 3 (Figure 10a), a few hours after the PEG was introduced, but it did not occur with AVD4 (Figure 10b). This isolated increase in the observed AVD1 values could be attributed to external noise, potentially caused by vibrations captured in the measurement environment.

It was previously noted that AVD4 offers a higher signal-to-noise ratio than AVD1. Therefore, due to its normalisation, the AVD4 index seems to attenuate noise.

The decrease in AVD values corresponds to a decrease in values that lie far from the diagonal of the COM matrix (see Figure 7e). Changes in intensity are lower at the end of the experiment. PEG was introduced in the sunflower nutrient medium during day 3. The introduction of PEG led to a gradual decay in AVD values, and therefore in biospeckle activity, for both sunflower realisations. The difference in values between day and night was also affected, with smaller differences following PEG addition, probably due to the continuous decrease.

This measured reduction in activity could be considered as the plant’s response to stress induced by PEG. In order to minimize damage related to this stress and its resources, the plant can deploy various physiological and biochemical mechanisms. The closing of the stomata allows the plant to limit transpiration and prevent water loss [49]. This stomatal closing directly affects photosynthesis, as it reduces the supply of CO2 necessary for this process [50]. Consequently, the plant decreases its metabolic activity [51], thus reducing the demand for energy and water resources, which also impacts the sap flow [52]. The water flow through the plant decreases, since less water is absorbed by the roots and lost by the leaves. The transport of nutrients and water within the plant is thus altered [53]. Moreover, when under stress, the diurnal and nocturnal activity of the plant decreases sharply. Stomatal closing during the day reduces transpiration and photosynthesis to similar levels similar as those at night, when these processes are naturally reduced in the absence of sunlight [54]. Finally, the day–night cycle, during which a plant is normally more active during the day for photosynthesis and less active at night, slows down under water stress. The plant maintains its stomata completely or partially closed during both day and night in order to minimise water loss, hence leading to a general reduction in metabolic activity and a blurring of the differences between day and night [55]. For this reason, biospeckle activity, measured through the AVD indicator, presents similar intensity levels. These mechanisms are implemented by the plant during water stress so as to keep up a certain level of activity.

### 3.3. Case Study for Plant Breeding: Application to a Tolerant Variety

**Figure 11:**
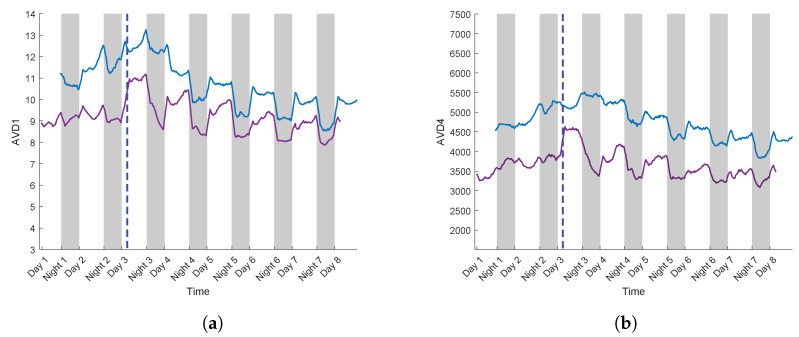
Biospeckle activity calculated by AVD with realisation 1 in purple and 2 in blue. Dotted lines correspond to PEG introduction with the normalized COM matrix $M_{ij,1}$ (**a**) and $M_{ij,2}$ (**b**).

In order to study the potential of this methodology for plant breeding, the same protocol as described above was applied to variety T, known to be tolerant to water stress. Figure 11a,b depict the evolution of AVD values for normalisations $M_{ij,1}$ (AVD1) and $M_{ij,2}$ (AVD4) of variety T, where the water constraint protocol was applied.

These two plants correspond to realisation 1 and 2 of variety T (Table 1), renowned for its resistance to water stress. As observed for control plants (Figure 9a,b), the differences between day and night were significant, with higher AVD values during the day.

Following the PEG addition on day 3, a progressive decrease in AVD values was observed for the two realisations under both normalisation methods.

For AVD1, the values between days 3 and 8 decreased by 5 for realisation 1, and by 3 for realisation 2. With AVD4, over the same period, AVD values dropped by 1524 for realisation 1, and by 728 for realisation 2.

At the end of the experiment, AVD values for variety T were higher than those for variety S for both normalisations (Figure 10a,b).

Unlike the observations on variety S (Figure 10a,b), after PEG addition on day 3, the AVD values differences between daytime and night-time conditions remained unchanged for both realisation of variety T (Figure 11a,b). Variety T maintained higher biospeckle activity levels than variety S after the addition of PEG, thus demonstrating a lower sensitivity to the stress induced by PEG. Renowned for its resistance to water stress, variety T can deploy adaptive mechanisms that allow it to regulate and maintain a more stable metabolic activity, particularly by consistent activity dynamics between day and night. These observations confirm the enhanced ability of variety T to adapt to water stress conditions.

### 3.4. Linear Regression Curve

In order to compare the impact of the PEG on both varieties and on the two types of normalisation, regression lines were calculated. For the realisation involving the introduction of PEG during the experiment, normalisation was performed using their highest value for AVD1 value and AVD4. Subsequently, a linear regression was calculated following the addition of PEG.

**Figure 12:**
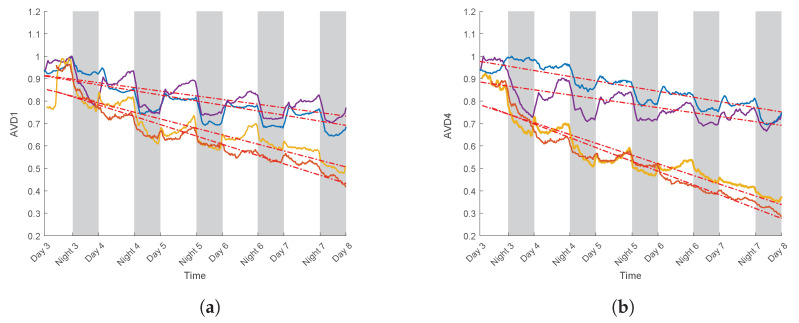
Value of AVD1 (**a**) and AVD4 (**b**) for realisations 1, 2, 3 and 4 (respectively purple, blue, yellow and orange) with their respective regression lines, after introduction of the PEG. Each curve is normalised by its highest value.

The below figures represent the values of AVD1 and AVD4 (Figure 12) with their associated linear regression lines, for the two studied varieties, after the addition of PEG. For each line, a slope coefficient was calculated (Table 3).

For AVD1 and AVD4 (Figure 12), a decrease in AVD values occurred following the addition of PEG. This was characterized by a negative coefficient of the regression lines (see Table 3). However, this decrease was more pronounced for variety S, with lower regression slope values than for variety T (Figure 12). In the case of AVD1 (Table 3) for variety T and realisations 1 and 2, the regression slopes were −0.82 and −0.70, respectively, while for variety S, they were −0.89 and −0.95 for realisations 3 and 4. This same trend was found with the AVD4 normalisation. For variety T and realisations 1 and 2, the regression slopes were −0.89 and −0.72, respectively, while for variety S, they were −0.93 and −0.96 for realisations 3 and 4, respectively.

It is also noteworthy that the AVD4 values between varieties S and T were more distinct. Indeed, the gap in the regression line coefficient values was more significant than for AVD1. As previously observed, AVD4 curves appeared to be less noisy. AVD4 therefore seems to be more suitable for distinguishing between the two varieties. These initial results are very encouraging. By measuring biospeckle and calculating numerical indicators, it has become possible to discriminate between varieties on the basis of their resistance to water stress at a relatively early stage.

## 4. Conclusions

This study highlighted the impact of water stress on biospeckle measurements, in particular through two varieties of sunflower known for their different behaviour regarding water stress. AVD values were calculated on two realisations for each variety submitted to the water stress protocol using PEG. The AVD indicator values of the sensitive variety fell sharply, in contrast to those of the tolerant variety. In addition, the day and night cycles, visible in AVD values, were strongly impacted for the sensitive variety after the onset of water stress.

In this study, the midrib was defined as the most noteworthy part of the leaf. It could be worthwhile to identify other areas of the leaf that could be potentially impacted by the onset of water stress, such as secondary veins or limbus. A spatial analysis of the results could then be carried out to examine the behaviour of biospeckle activity in different parts of of the leaf. These approaches would enable an in-depth study of biospeckle activity and its variations across the leaf, thus providing a better understanding of the physiological processes taking place locally.

So far, these results are promising, highlighting the impact of PEG use on the studied variables. However, to strengthen the reliability and generalisability of these results, additional measurements should be conducted. The stomatal conductance of leaves can provide us with information on transpiration, stomatal opening, and the efficiency of photosynthesis. Hydraulic conductivity, on the other hand, gives us insights into how easily water and solutes move through plant tissues, offering clues about the health and function of the conducting vessels in the leaves. These new measurements would not only consolidate the observed trends but could provide insight into the possible variations among different varieties or experimental conditions.

Biospeckle imaging could be a useful tool for identifying typical genotype profiles in the context of water stress, and could be extended to a study involving a larger number of genotypes. These initial results are encouraging for a more widespread use as a phenotyping tool in other cases of varietal selection, such as resistance to biotic stress.

## Figures and Tables

**Table 1:** Measurement listing: temporal information and experimental conditions.

Realisation	Experiment Conditions	Variety	Start of Measurements	End of Measurements	PEG Introduction
1	PEG	T	04-Aug-2023 09:43:16	11-Aug-2023 10:28:28	06-Aug-2023 10:28:40
2	PEG	T	15-May-2023 20:11:34	25-May-2023 10:10:34	18-May-2023 10:02:38
3	PEG	S	16-Mar-2021 10:15:36	23-Mar-2021 08:51:16	18-Mar-2021 14:45:48
4	PEG	S	29-Jun-2021 11:45:38	06-Jul-2021 09:01:48	01-Jul-2021 10:45:42
5	Control	S	24-Feb-2023 10:41:00	03-Mar-2023 10:21:00	None
6	Control	S	16-Feb-2023 15:20:00	23-Feb-2023 15:00:12	None
7	Reference	Inert object	27-Jan-2023 16:12:42	30-Jan-2023 07:32:46	None

**Table 3:** Values of the slopes of the linear regression of realisations 1, 2, 3, and 4 after the introduction of the PEG, for AVD1 and AVD4.

Realisations	Variety	Slope after PEG	
		AVD1	AVD4
1	T	−0.82	−0.89
2	T	−0.70	−0.72
3	S	−0.89	−0.93
4	S	−0.95	−0.96

## Data Availability

Data will be made available on request.
